# Choked on Potato and Beef

**Published:** 1873

**Authors:** 

**Affiliations:** Shadeland, Ga.


*Messrs. Editors,*—Did you ever eat a cold sweet potato? If so, you got choked, more or less, or at least came very near it. I say this from a lively recollection of my country schoolboy days, and the cold dinners of those days, in which cold potatoes and difficult digestion entered so largely. Now this is brought to mind by a case that lately came to hand. True, there is nothing in the case particularly striking. Certainly nothing startling in character or novel in treatment. But I nevertheless present it to show my country brethren who may be deficient in professional "armamentarium," what common sense and a little nerve may do toward supplying these deficiencies, when guided by the tutillage of the presiding divinities, anatomy and physiology. And further, I will not conceal the fact that my ambition is somewhat stirred to share, in some small degree, the compliment so kindly and gracefully bestowed by that admirable writer and surgeon, Dr. Battey, on M.D.'s of the mountain region—telling how fruitful they are in expedients to meet emergencies—for instance, using kitchen pot-hooks, instead of obstetrical blunt hooks, to relieve parturient sufferers. But to the case:

On the 21st of September, at 5 o'clock P.M., responding to an "alarm at the door," I met a long, lank, lean Caucasian, who anxiously inquired for an "elastic tube," which I soon found he wanted to remove cold potatoes and beef from the œsophagus, which had lodged there while eating his supper the evening before. He said he had been to the city of——, trying the drug stores and doctors, but could get neither tube or relief from the "tater" and beef. He further said he had drunk water repeatedly, but it neither pushed on the potato, or brought it back in the refluent tide. There was no pain, no dyspnœa, only a sense of fullness at the point of obstruction, near the cardiac orifice. He was sadly chapfallen at not finding an "elastic tube," which he seemed to have his heart very much set upon. After satisfying him that such an implement was not needed, I got a rattan umbrella rib, to make a probang. Not that I preferred rattan to several other things, but because it was at hand and nearly made to hand. After polishing the staff, securely attaching a piece of sponge, and oiling all, the rude instrument was introduced slowly and steadily, meeting no resistance until the edibles were reached, and but little then.

But, Messrs. Editors, it was like deep sea soundings to reach that vegetable. Just to think of it, a man between six and seven feet *long*, and, from my explorations with an umbrella rib, it seemed that his upper half was entirely too long for the rest of him. But, in spite of disappearing rattan, I boldly pushed on, assured that the deepest sea has a bottom, and the longest œsophagus *another end* somewhere below. Perfect success verified the soundness of my reasoning, and crowned the boldness of my efforts in the removal of the offending mass. A drink of water now did not regurgitate as before, but went on traversing the usual round to the other emunctories.

Thus ends a homely narrative, as abruptly as it began, from your country friend, A. COUVERT, M.D.

*Shadeland, Ga., September,* 1873.

